# Investigating the Resistance Mechanism of Wheat Varieties to Fusarium Head Blight Using Comparative Metabolomics

**DOI:** 10.3390/ijms24043214

**Published:** 2023-02-06

**Authors:** Yifan Dong, Xiaobo Xia, Dawood Ahmad, Yuhua Wang, Xu Zhang, Lei Wu, Peng Jiang, Peng Zhang, Xiujuan Yang, Gang Li, Yi He

**Affiliations:** 1 CIMMYT-JAAS Joint Center for Wheat Diseases, The Research Center of Wheat Scab, Jiangsu Academy of Agricultural Sciences, Nanjing 210014, China; 2 Department of Plant Pathology, College of Plant Protection, Nanjing Agricultural University, Nanjing 210095, China; 3 School of Agriculture, Food and Wine, Waite Research Institute, The University of Adelaide, Waite Campus, Adelaide, SA 5064, Australia

**Keywords:** metabolomics, wheat, Fusarium head blight, fungistatic, gene expression, resistance, susceptibility

## Abstract

Fusarium head blight (FHB) is primarily caused by *Fusarium graminearum* and severely reduces wheat yield, causing mycotoxin contamination in grains and derived products. *F. graminearum*-secreted chemical toxins stably accumulate in plant cells, disturbing host metabolic homeostasis. We determined the potential mechanisms underlying FHB resistance and susceptibility in wheat. Three representative wheat varieties (Sumai 3, Yangmai 158, and Annong 8455) were inoculated with *F. graminearum* and their metabolite changes were assessed and compared. In total, 365 differentiated metabolites were successfully identified. Amino acids and derivatives, carbohydrates, flavonoids, hydroxycinnamate derivatives, lipids, and nucleotides constituted the major changes in response to fungal infection. Changes in defense-associated metabolites, such as flavonoids and hydroxycinnamate derivatives, were dynamic and differed among the varieties. Nucleotide and amino acid metabolism and the tricarboxylic acid cycle were more active in the highly and moderately resistant varieties than in the highly susceptible variety. We demonstrated that two plant-derived metabolites, phenylalanine and malate, significantly suppressed *F. graminearum* growth. The genes encoding the biosynthetic enzymes for these two metabolites were upregulated in wheat spike during *F. graminearum* infection. Thus, our findings uncovered the metabolic basis of resistance and susceptibility of wheat to *F. graminearum* and provided insights into engineering metabolic pathways to enhance FHB resistance in wheat.

## 1. Introduction

Wheat (*Triticum aestivum* L.) is an important food crop that provides starch, protein, and dietary fiber to >40% of the global population [1]. With the soaring human population worldwide, the demand for wheat yield keeps increasing. However, the annual worldwide wheat production is constantly threatened by various diseases [2]. One of the most disastrous disease, Fusarium head blight (FHB or scab), is caused by one or more pathogenic *Fusarium* species, such as *Fusarium culmorum* (*Fc*), *F. avenaceum* (*Fa*), and *F. graminearum* (*Fg*). FHB causes yield losses of up to 50% and contaminates grains with mycotoxins such as deoxynivalenol (DON), leading to poor quality [2]. During infection, mycotoxins can stably accumulate within plant tissues and cells; undermine plant signaling, metabolism, and development; and facilitate the FHB proliferation and spread among wheat spikelets [3].

In cereals, FHB resistance is a highly complex quantitative trait controlled by multiple loci, environment, and their interactions [4,5]. Over 600 quantitative trait loci (QTLs) contributing to FHB resistance have been identified across the 21 wheat chromosomes [5]. However, only seven QTLs (*Fhb1*–*Fhb7*) have been formally assigned with a gene name, and two genes, *Fhb1* and *Fhb7*, have been cloned via QTL mapping [6]. *Fhb1*, encoding a histidine-rich calcium-binding protein, was first identified in a resistant wheat variety, Sumai 3, and has been recognized as the most effective and robust QTL for breeding [7,8]. *Fhb7* originates from *Thinopyrum elongatum* and encodes a glutathione S-transferase that can detoxify trichothecene [9]. Revealing the molecular mechanisms underlying FHB resistance may provide an important basis to improve FHB resistance in wheat. Previously, a transcriptomics analysis has also been applied to understand the molecular mechanisms underlying FHB resistance in wheat and identified a number of differentially expressed genes/transcripts in different cultivars [10,11,12,13,14,15,16,17].

Despite the progress made in mapping and cloning QTLs, along with deciphering gene-expression related changes, the wheat host resistance mechanisms against FHB remain poorly understood [18]. Thus, a metabolomics study may open a new avenue for probing plant–pathogen interactions during FHB infection and help develop a strategy to tailor future-ready FHB-resistant wheat varieties [19,20]. The metabolomic analysis is a novel tool to identify the biochemical fingerprints for host–pathogen interactions by quantifying metabolites in a targeted or nontargeted approach [21,22,23]. Gunnaiah and Kushalappa [18] proposed that metabolites are considered factors interfering with *F. graminearum* infection in different wheat varieties. The biochemical profiling of FHB-infected cereals can assist in deciphering the mechanism underlying FHB resistance [24]. This can be achieved by analyzing the metabolome in genotypic and phenotypic contexts to isolate specific metabolites critical for FHB resistance [21,25]. However, not many studies have been conducted on wheat to identify global metabolic changes during wheat Fusarium head blight infection. Therefore, in this study, highly resistant, moderately resistant, and highly susceptible wheat varieties were used for global metabolomics investigation to understand how differentiated metabolic responses during FHB infection contribute to resistance in three commercially grown varieties in China.

## 2. Results

### 2.1. Evaluation of FHB Resistance among Wheat Varieties

**Figure 1:**
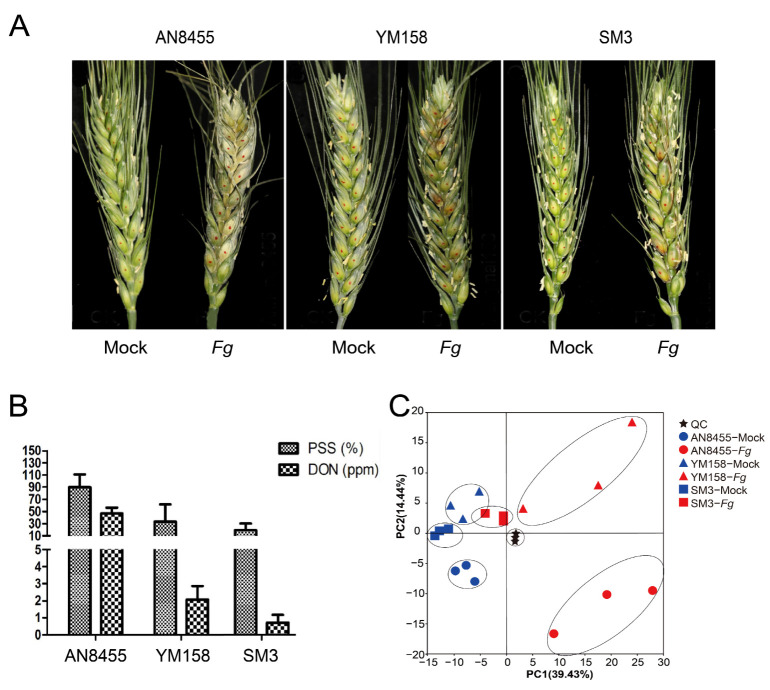
Resistance to *F. graminearum* is variety-dependent. (**A**) A representative picture of mock- and *Fg*-inoculated wheat heads belonging to three wheat cultivars: AN8455, YM158, and SM3. Pictures were taken 4 days post-inoculation. (**B**) Percentage of scabbed spikelets (PSS) and deoxynivalenol (DON) content in infected spikelets of different wheat varieties. (**C**) Principal component analysis (PCA) diagram of detected metabolites. The stars represent quality controls (QC). Red indicates samples with *Fg* treatment, and blue indicates mock inoculated samples.

To assess the susceptibility of different wheat varieties to *F. graminearum*, we conducted spikelet infection assays on the highly susceptible variety (SV) Annong 8455 (AN8455), moderately resistant variety (MRV) Yangmai 158 (YM158), and highly resistant variety (RV) Sumai 3 (SM3). The spikelets of AN8455 withered severely following *F. graminearum* infection, YM158 exhibited a less severe withering, whereas the spikelets of SM3 demonstrated only slight withering (Figure 1A). Consistently, SM3 showed highest resistance, followed by YM158, while AN8455, on the other hand, was the most susceptible variety, and maximum amount of DON was accumulated in AN8455 (Figure 1B).

The spikelets of *Fg*-inoculated (4 days post-inoculations; dpi) plants were utilized to analyze the metabolomes. In total, 365 metabolites were detected in this study (Supplementary Table S1). All detected metabolites were used for the principal component analysis (PCA), in which three quality controls were clustered together, confirming the reliability of the data (Figure 1C). With 14.44% variance for principal component two, the variance within the biological replicates of each group was much smaller than between the infected and mock groups (39.43% variance for principal component one) (Figure 1C). Therefore, the infected and mock samples were distinguished according to their respective metabolite profiles. Remarkably, the variance between the infected SM3 and mock samples was much lower that between AN8455 and YM158, indicating different degrees of metabolic responses among varieties.

### 2.2. Dynamic Changes in Metabolites among Different Wheat Varieties during FHB Infection

**Figure 2:**
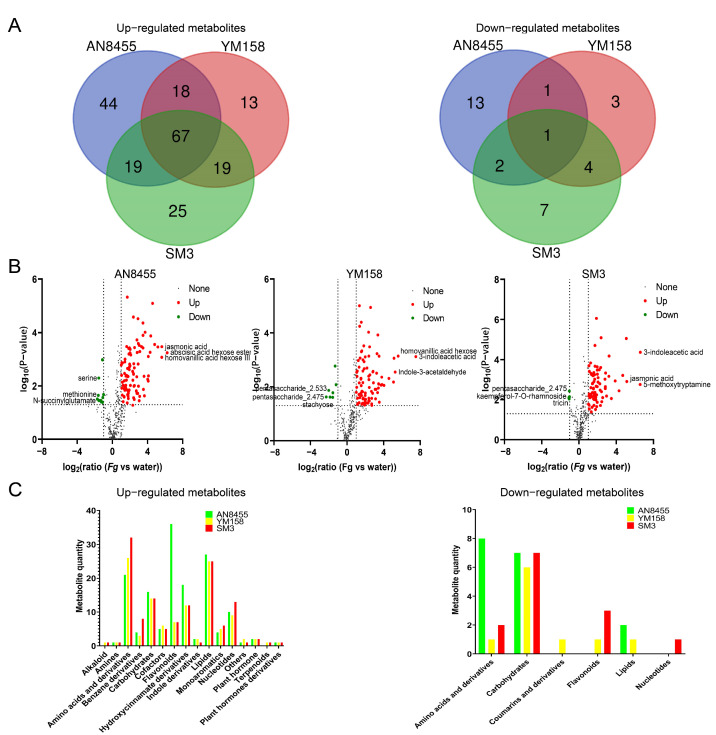
Analysis of metabolomic data. (**A**) Venn diagram of upregulated and downregulated metabolites in the three varieties: AN8455, YM158, and SM3. (**B**) The volcano plots of metabolites. Metabolites that are upregulated and downregulated by more than two times are marked. The upregulated metabolites are marked in red, and the downregulated metabolites are marked in green. (**C**) Column statistical chart of the types and quantities of upregulated and downregulated metabolites in the three varieties.

Metabolites were differentially upregulated and downregulated in AN8455, YM158, and SM3 (Supplementary Figure S1). Specifically, among the upregulated metabolites, 67 metabolites were shared by three varieties, 18 metabolites were shared by AN8455 and YM158, 19 metabolites were shared by AN8455 and SM3, and 19 metabolites were shared by YM158 and SM3 (Figure 2A; Table 1). Among the downregulated metabolites, only one metabolite was shared by the three varieties, one metabolite was shared by AN8455 and YM158, two metabolites were shared by AN8455 and SM3, and four metabolites were shared by YM158 and SM3 (Figure 2A; Table 1). There were 44 uniquely upregulated and 13 downregulated metabolites in AN8455, 13 uniquely upregulated and 3 downregulated metabolites in YM158, and 25 uniquely upregulated and 7 downregulated metabolites in SM3.

The detected metabolites of each variety were used to generate volcano plots. Overall, there were more upregulated metabolites than downregulated ones in all the three varieties (Figure 2B). Histogram statistics were used to characterize different types of metabolites in AN8455, YM158 and SM3 (Figure 2C). Amino acids and derivatives, carbohydrates, flavonoids, hydroxycinnamate derivatives, lipids, and nucleotides constituted the major changes in response to fungal infection. However, these metabolites exhibited changing trends in wheat varieties with different resistance levels. For example, AN8455 was found to have the largest number of downregulated amino acids and derivatives, whereas SM3 showed the most upregulated amino acids (Figure 2C).

### 2.3. Response of Metabolites in Catabolic Processes

**Figure 3:**
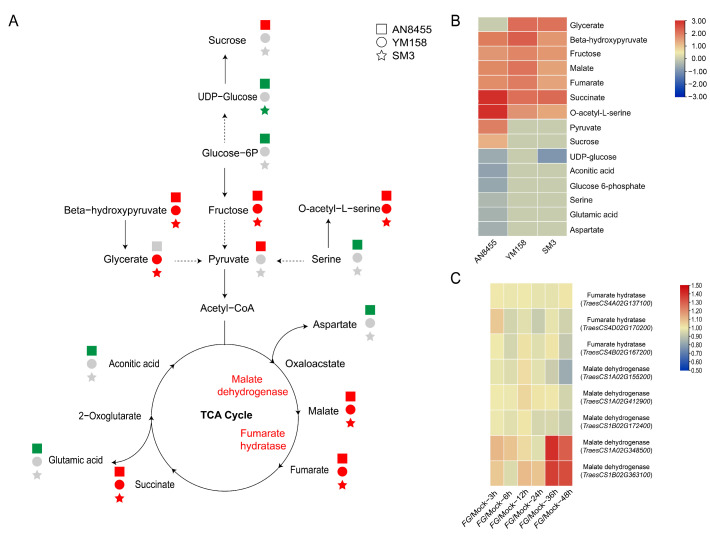
Metabolites that participated in catabolic processes. (**A**) Schematic diagram of catabolic processes. The squares, circles, and stars represent AN8455, YM158, and SM3, respectively. Red, green, and gray shapes represent upregulated expression, no obvious change, and downregulated expression, respectively. (**B**) Radio value (*Fg* vs. Mock) of metabolites involved in catabolic processes. (**C**) Gene expression heatmap of enzymes involved in catabolic processes, sourcing from WheatGene (http://wheatgene.agrinome.org/ (accessed on 9 July 2022)).

Phenylpropanoids, carbohydrates, terpenoids, amino acids and derivatives, and fatty acids in infected wheat heads have been previously implicated as potential contributors to FHB resistance [19]. We first analyzed the metabolites of the catabolic processes (Figure 3A), then radio value (*Fg* vs. Mock) was used to generate a heatmap that depicted the variations among the three varieties (Figure 3B). The tricarbonic acid cycle (TCA cycle) constitutes the central pathway for energy generation in the cells, and multiple other biochemical pathways are connected to the TCA cycle [26]. We observed that six metabolites were significantly upregulated of all the three varieties, including malate, fumarate, and succinate of the TCA cycle, and fructose, beta-hydroxypyruvate, and O-acetyl-L-serine (Figure 3A,B). Five metabolites, including aconitic acid, aspartate, glutamic acid, serine, and glucose-6P, were only significantly downregulated in SV AN8455 but remained unchanged in MRV YM158 and RV SM3 (Figure 3A,B). We next examined the expression of relevant genes following *F. graminearum* infection (Supplementary Table S2) [27]. Two genes, *TraesCS1A02G348500* and *TraesCS1B02G363100*, encoding malate dehydrogenases, were upregulated after *F. graminearum* infection (Figure 3C).

### 2.4. Response of Nucleotide Metabolism

**Figure 4:**
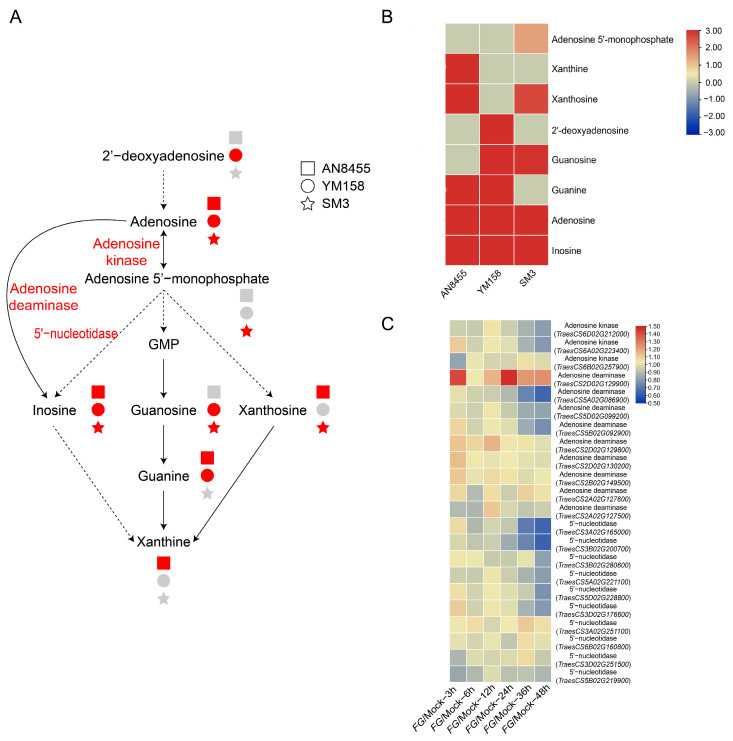
Metabolites that participated in nucleotide metabolism. (**A**) Schematic diagram of catabolic processes. The squares, circles, and stars represent AN8455, YM158, and SM3, respectively. Red, green, and gray shapes represent upregulated expression, no obvious change, and downregulated expression, respectively. (**B**) Radio value (*Fg* vs. Mock) of metabolites involved in catabolic processes. (**C**) Gene expression heatmap of enzymes involved in nucleotide metabolism, sourcing from WheatGene (http://wheatgene.agrinome.org/ (accessed on 9 July 2022)).

*F. graminearum* infection is reported to induce nucleotide production [28]. In this study, adenosine and inosine were upregulated in all the three wheat varieties (Figure 4A,B). Inosine and xanthine upregulation was more pronounced in AN8455 than in YM158 and SM3, 2′-teoxyadenosine, adenosine, and guanine upregulation was more pronounced in YM158 than in AN8455 and SM3, and adenosine 5′-monophosphate upregulation was more pronounced in SM3 than in YM158 and AN8455 (Figure 4B). Genes encoding adenosine deaminase, 5′-nucleotidase, and adenosine kinase (Supplementary Table S3) were identified, but only one adenosine deaminase gene, *TraesCS2D02G129900,* was upregulated following *F. graminearum* infection (Figure 4C) [27]. Several genes encoding adenosine deaminase and 5′-nucleotidase were downregulated in response to *F. graminearum* (Figure 4C), suggesting the dynamic gene expression during FHB infection to regulate nucleotide metabolism in wheat.

### 2.5. Response of Amino Acid and Derivatives

**Figure 5:**
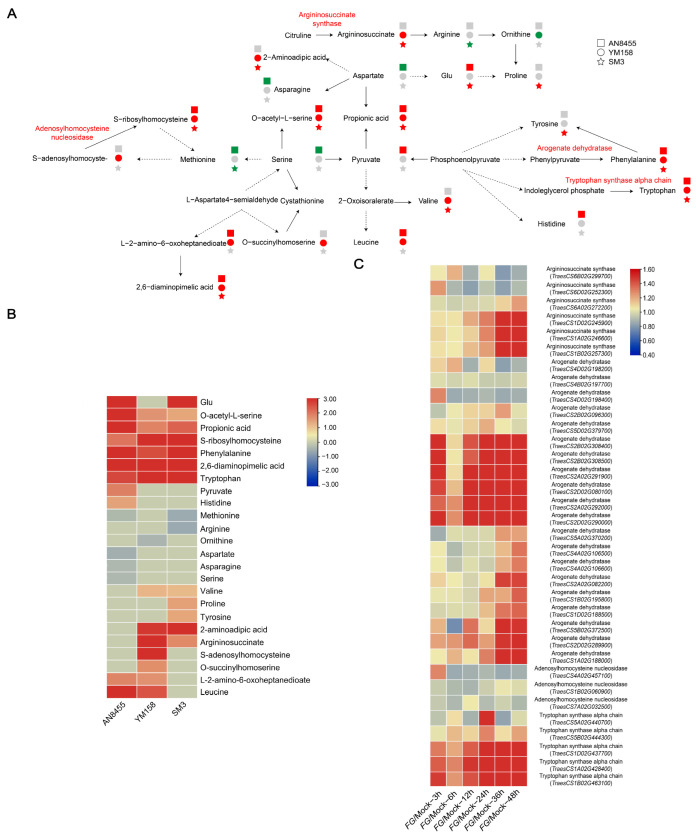
Amino acid and derivatives. (**A**) Schematic diagram of amino acid biosynthesis. The squares, circles, and stars represent AN8455, YM158, and SM3, respectively. Red, green, and gray shapes represent upregulated expression, no obvious change, and downregulated expression, respectively. (**B**) Radio value (*Fg* vs. Mock) of amino acid and derivatives. (**C**) Gene expression heatmap of enzymes involved in amino acid biosynthesis, sourcing from WheatGene (http://wheatgene.agrinome.org/ (accessed on 9 July 2022)).

Amino acids, such as proline and alanine, have been reported to enhance FHB resistance [29]. In this study, proline and tyrosine were significantly upregulated in SM3, and valine, 2-aminoadipic acid, and argininosuccinate were upregulated in both SM3 and YM158 (Figure 5A). Six metabolites, including tryptophan, phenylalanine, propionic acid, O-acetyl-L-serine, S-ribosylhomocysteine, and 2,6-diaminopimelic acid, were significantly upregulated in all three varieties. Among these metabolites, tryptophan, proline, and tyrosine were more pronounced in SM3 than in AN8455 and YM158, and propionic acid, glutamate, leucine, histidine, 2,6-diaminopimelic acid, and O-acetyl-L-serine were more pronounced in AN8455 than in YM158 and SM3 (Figure 5B). Most of the genes encoding these enzymes except adenosylhomocysteine nucleosidase were upregulated following *F. graminearum* infection (Figure 5C; Supplementary Table S4) [27].

### 2.6. Response of Antifungal Metabolites

Metabolomics can provide a snapshot of plant metabolism in response to a wide range of biotic stimuli. Numerous compounds potentially associated with plant–pathogen interactions have been identified [30]. Based on the previously reported functions, 16 antifungal and 21 immunomodulatory compounds were found in this study (Table 2). Among the antifungal metabolites, phenylalanine, spermidine, tryptophan, maleic acid, fumarate, fructose, nicotinamide, and cinnamic acid were upregulated in the three varieties, and benzoic acid, glucosamine, and kaempferol-3-O-glucoside II were upregulated in SM3 (Table 2). Among the metabolites regulating plant immunity, agmatine, succinate, and jasmonic acid were upregulated in the three varieties; proline, argininosuccinate, gamma-aminobutyrate, m-salicylic acid, and guanosine were upregulated in only SM3; and asparagine and raffinose were downregulated in AN8455.

### 2.7. Phenylalanine and Malate Suppress the Growth of F. graminearum

**Figure 6:**
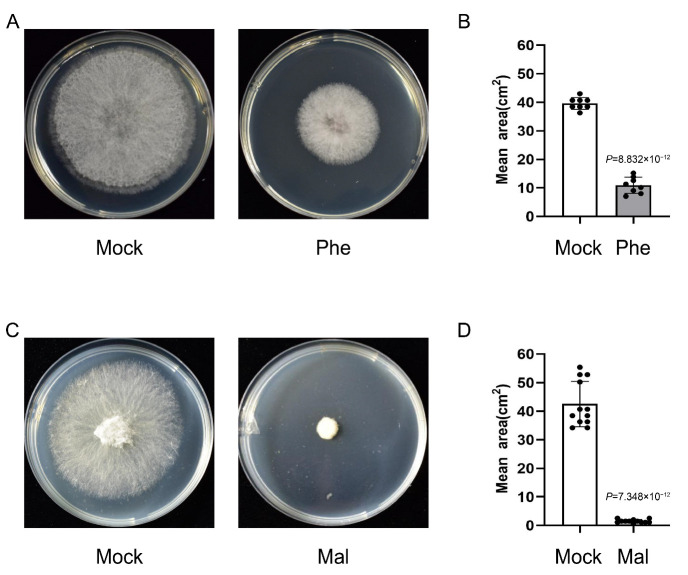
Antifungal effects of phenylalanine and malate. (**A**) Growth of the *F. graminearum* strain Fg1312 on potato dextrose agar (PDA) cultures with 5 mM Phenylalanine or mock as control at 25 °C for 3 d. (**B**) Statistical analysis of colony growth area of the Fg1312 strains on respective media. Error bars represent standard deviation from 8 independent experiments. (**C**) Growth of the *F. graminearum* strain Fg1312 on potato dextrose agar (PDA) cultures with 5 mM malate or mock as control at 25 °C for 3 d. (**D**) Statistical analysis of colony growth area of the Fg1312 strains on respective media. Error bars represent standard deviation from 12 independent experiments.

Several natural metabolites potentially contribute to FHB resistance by suppressing pathogen growth. For example, flavonoids are well known for inhibiting fungal spore germination and restraining mycelium hyphae elongation [19]. To mine new metabolites that may inhibit *F. graminearum* growth, a selection of metabolites related to antifungal and immunity, including glutamine, γ-aminobutyric acid, phenylalanine, proline, leucine, and malate, were tested for in a fungistatic experiment (Figure 3 and Figure 5). Interestingly, phenylalanine (5 mM) and malate (5 mM) exhibited a strong inhibiting effect on *F. graminearum* growth (Figure 6A,C). By comparing the average growth area of hyphae, we found that the malate has a superior ability to limit hyphae growing and spreading (Figure 6B,D). The effects of 5 mM glutamine, 5 mM proline, 5 mM γ-aminobutyric acid, and 1 mM leucine were also tested, and no significant suppressing effects were observed (Supplementary Figure S2).

**Figure 7:**
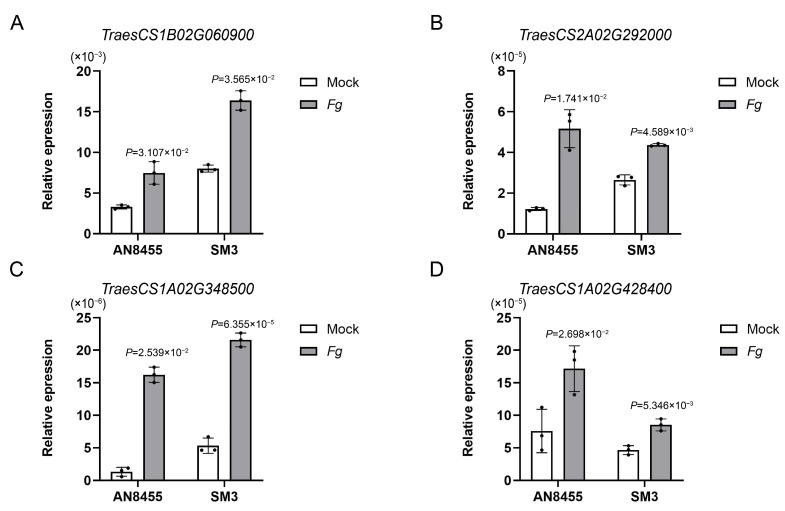
Upregulation of genes involved in the metabolic pathways after *F. graminearum* infection. (**A**) Histogram of adenosylhomocysteine nucleosidase gene expression in AN8455 and SM3. The wheat *tubulin* gene was used as the internal control, and a *t*-test was used to compare significant differences. (**B**) Histogram of arogenate dehydratase gene expression in AN8455 and SM3. (**C**) Histogram of malate dehydrogenase gene expression in AN8455 and SM3. (**D**) Histogram of tryptophan synthase alpha chain gene expression in AN8455 and SM3. Error bars show the standard deviation. Data are presented as mean ± SD (n = 3).

We found that four genes, which encode adenosylhomocysteine nucleosidase, arogenate dehydratase, malate dehydrogenase, and tryptophan synthase alpha chain, were upregulated following infection in AN8455 and SM3, as revealed by qRT-PCR. Genes encoding arogenate dehydratase and malate dehydrogenase are required to synthesize phenylalanine and malate. The other two genes, adenosylhomocysteine nucleosidase and tryptophan synthase, participate in the synthesis of s-ribosylhomocysteine and phenylalanine (Figure 7A–D).

## 3. Discussion

Addressing FHB in commercially essential food crops, such as wheat and maize, is crucial for food security and public health. Identifying host resistance factors can assist in combating FHB. A diverse set of metabolites have been identified which potentially suppress FHB symptoms in wheat [19]. The number of identified metabolites varied in different studies depending upon the genetic material and analytical strategy utilized, for example, 10 in a HNMR study and >500 in the survey based on UHPLC–QTOF/MS [31,32]. In this study, metabolome profiling of *F. graminearum* inoculated three Chinese commercially wheat varieties, AN8455, YM158, and SM3, which exhibit varying levels of FHB resistance, was conducted using UHPLC–MS/MS technology. A total of 365 compounds were detected from the methanol extracts of the three varieties. These chemical compounds were quantitatively analyzed and compared in the context of responding to *F. graminearum* infection.

Consistent with the findings of a previous report [19], flavonoids, hydroxycinnamate derivatives, amino acids and derivatives, carbohydrates, lipids, and a new group of nucleotides were significantly altered during the fungal infection in highly susceptible variety AN8455, moderately resistant variety YM158, and highly resistant variety SM3. However, their altered profiles depend on the genotype of the wheat variety. Particularly, phenylpropanoid compounds, giving rise to metabolites, such as flavonoids, lignans, phenylpropanoid esters, and hydroxycinnamic acid amides, are believed to be involved in plant resistance to fungal pathogens [33]. In this study, we identified 88 flavonoids, 15 benzene derivatives, and 29 hydroxycinnamate derivatives, among which, 37, 8, and 20 were, respectively, significantly altered in the AN8455, YM158 and SM3. The flavonoid compound naringenin, a reportedly efficient inhibitor of *F. graminearum* growth in vitro [34], was upregulated by 12.55-, 2.89-, 3.53-fold in AN8455, YM158, and SM3, respectively; o-coumaric acid, a hydroxycinnamate derivative, which reduce pathogen advancement as phytoalexins and cell wall strengthening agent [33], was upregulated by 7.74-, 5.41-, 3.71-fold in AN8455, YM158, and SM3 respectively. However, apparently, more phenylpropanoid compounds were significantly upregulated in AN8455 than in YM158 and SM3. Thus, these metabolites may be the basis resistance but not the main reasons for the different resistance of the three varieties. This also indicates that other metabolites promoting resistance are needed in resistant genotypes in addition to phenylpropanoid compounds.

FHB resistance in wheat is reportedly associated with the phenylpropanoid, terpenoid, and fatty acid pathways, which are involved in plant defense signaling, antimicrobial activity, and cell wall thickening [19,26]. We further found that differentially accumulated metabolites were primarily concentrated in three metabolic pathways: carbon metabolism, nucleotide metabolism, and amino acid biosynthesis. The metabolic profiling of wheat spikelets revealed that sugars could account for wheat resistance to *F. graminearum* and DON accumulation [19]. The TCA cycle is the central energy-generating pathway in the cell. As a signaling molecule, sucrose participates in numerous plant metabolic processes, is essential for plant growth, and can activate the plant immune response [35]. Exogenous pretreatment with fructose can enhance the innate immunity of *Arabidopsis thaliana* [36]. Fumarate accumulation in plants integrates immune and metabolic circuits [37]. The malate released by *Arabidopsis* protects root tips from toxicity and recruits beneficial root bacteria to induce plant immunity, thereby enhancing the tolerance of plants to some environmental stress factors [38]. We identified 44 carbohydrates, and the elevations of succinate, malate, and fumarate, together with the upregulation of malate dehydrogenase gene *TraesCS1A02G348500* indicate enhancement of the TCA cycle in all three varieties. Amino acid metabolism and the biosynthesis pathway participate in wheat resistance to FHB [29]. Proline and alanine could inhibit *F. graminearum* occurrence to a certain extent and significantly improve *F. graminearum* resistance in susceptible varieties [29]. Exogenous pretreatment with phenylalanine can induce a broad-spectrum immune response in plants [39]. DON produced by *F. graminearum* can increase the level of amino acids in wheat, which leads to the activation of a defense response in wheat [40]. In this study, 70 amino acids were upregulated, and the upregulation of many amino acids such as cycloleucine, actinonin, proline, reduced glutathione, argininosuccinate, cysteine, tyrosylglutamate, N-acetylcitrulline, and 5-methoxytryptamine was higher in SM3 than in AN8455, consistent with the highly resistant nature of SM3 to FHB. Nucleotides are essential for life; however, their role in FHB resistance remains unclear. We identified 27 nucleotides, some of which were highly upregulated. For example, thymidine was upregulated at 28.04-, 33.95-, and 19.80-fold in AN8455, YM158, and SM3, respectively.

Several plant metabolites have been identified to have antifungal, signaling, and cell wall enforcement properties. In this study, we identified 16 metabolites with antifungal properties and 21 with plant immunity regulatory properties. Among the metabolites possessing antifungal properties, phenylalanine, spermidine, tryptophan, maleic acid, fumarate, fructose, nicotinamide, and cinnamic acid were upregulated in the three varieties, likely functioning to resist pathogen infection. Furthermore, benzoic acid, glucosamine, and kaempferol-3-O-glucoside II were significantly upregulated in only SM3, indicating that these factors might confer high resistance in resistant genotypes. Many metabolites can be used as fungistatic agents to suppress the mycelial growth of fungal pathogens [41]. One of the mechanisms of plant resistance is the accumulation of metabolites with high antifungal activity, such as alkaloids and isoflavones [42]. The plant glucosinolate pathway is a broad-spectrum antifungal defense response in plants [43]. In the present study, we found that phenylalanine, which is involved in amino acid biosynthesis, and malate, which is involved in carbon metabolism, can suppress the growth of *F. graminearum* mycelium. This finding indicates that phenylalanine and malate might be used as green fungistatic agents in the future to provide resistance to FHB. Additionally, the expression of several genes encoding key metabolic enzymes, such as malate dehydrogenase and arogenate dehydratase, was also found to be upregulated in the wheat spikelet after infection.

Phytohormones are essential signaling molecules and play crucial roles in controlling the expression of downstream defense genes and physiological reactions against various stresses [44]. Abscisic acid (ABA) is associated with FHB susceptibility [45,46,47]. The salicylic acid (SA) signal pathway is associated with FHB resistance at the early infection stage [48]. Conversely, jasmonic acid (JA) promotes the infection by constraining the SA signaling pathway during the early stage of infection and promotes resistance during the later stages of infection [19,31]. The upregulation of ABA in AN8455 was higher than in YM158 and SM3, which might be a reason for susceptibility in AN8455. SA was only significantly upregulated in SM3, and JA was 49.43-, 35.2-, and 35.14-fold upregulated in the three varieties. Furthermore, 3-indoleacetic acid (IAA) was 17.24-, 184.78-, and 96.02-fold upregulated in highly susceptible variety AN8455, moderately resistant variety YM158, and highly resistant variety SM3, respectively. However, there are few reports regarding the action of IAA in wheat–FHB interactions [49]. Future studies are warranted to understand the effect of IAA on wheat–*F. graminearum* interactions.

## 4. Materials and Methods

### 4.1. Plant Material

Three Chinese varieties, including YM158, AN8455, and SM3, were used in this study. Seedlings of these three varieties were grown in Petri dishes and later transferred to pots to be grown in a growth room at the Jiangsu Academy of Agricultural Sciences, Nanjing, China at a relative humidity of 70% and a 16 h photoperiod at 22 °C (light) and 18 °C (dark) conditions. Approximately 10 μL of the fungal suspension in mung bean soup (1 × 10^6^ conidia per mL) of *F. graminearum* strain Fg1312 was injected into the central spikelet (for testing the FHB resistance level) or all the spikelets in a spike (for the metabolites analysis) at early anthesis. The control plants were injected with 10 μL of water. The inoculated spikes were covered with a plastic bag for 2 days to meet the moisture requirement for fungal infection [50]. The wheat spikelets were harvested on day 4, lyophilized for 48 h, and stored at −80 °C for further metabolomics analysis. At least 10 spikelets were used for phenotypic observation and 3 spikes for mass spectrometry. The percentage of scabbed spikelets (PSS) were recorded for FHB phenotyping and the spikelets were harvested for DON measurement 21 days post-inoculations. DON measurement was performed using DON Elisa Kit (Solarbio) according to the manufacturer’s instructions.

### 4.2. Compound Extraction

The inoculated spikelets from AN8455, YM158 and SM3 were grinded into fine powder. A total of 20 mg of the powder from each sample was added to a fresh 2-mL EP tube. Then 1 mL of pre-cooled methanol was added to the powder, vortexed for 3 min, sonicated for 10 min, and centrifuged at 12,000× *g* for 10 min. The supernatant was transferred to a new 1.5-mL EP tube and centrifuged at 12,000× *g* for 10 min, and 200 µL of the supernatant was drawn into a glass liner. The inner liner was placed in a vial and then used for ultra-high performance liquid chromatography–quadrupole time-of-flight mass spectrometry/mass spectrometry analysis and data collection [51].

### 4.3. Chromatography and Mass Spectrometry

Ultra-high-performance liquid chromatography was performed on the Agilent’s 1290 Infinity II LC^TM^ system using an Agilent eclipse-plus C18 column (150 × 3.0 mm, I.D., 1.8 µm), and the column temperature was set as 40 °C [52]. The mobile phase consisted of A (0.1% carboxylic acid aqueous solution) and B (100% acetonitrile). The elution gradient of the mobile phase was as follows: 0 min (98.0% A), 1.0 min (98% A), 5.0 min (60% A), 12.0 min (30% A), 15.0 min (5% A), and 20.0 min (5% A). The flow rate of the mobile phase was 0.40 mL/min with a volume of 1.00 µL.

Mass spectrometry was performed using Agilent’s 6545 Q-TOF mass spectrometer detector. The full scan mode recorded data with a mass-to-charge ratio between 50 and 1000 m/z at a scanning rate of 2 spectra/sec. The electrospray ion source used the positive and negative cathode ionization modes, with a capillary voltage of 3.5 kV, a nozzle voltage of 500 V in the positive ion mode and 1500 V in the negative ion mode, and a fragment voltage of 110 V. The nebulizer pressure was 45 psi, and the sheath gas flow rate was 8 L/min. The collision voltages for acquiring the secondary mass spectra were 10 V, 20 V, and 40 V [51,52].

### 4.4. Identification of Compounds and Extraction of Peak Area

Metabolite identification and peak area extraction were performed as described previously [53]. Briefly, the metabolites were annotated by searching the Personal Compound Database and Library (PCD/PCDL) [54], and the Massbank [55] and Metlin [56] databases based on two criteria: (1) the difference between the observed mass and the theoretical mass was less than 5 ppm; (2) the main feature of the observed MS/MS spectrums was the same to that in literatures or database. Data acquisition, metabolite annotation and peak area extraction were performed with the Agilent software of MassHunter Acquisition 7.0, MassHunter Qualitative 7.0 and Mass Profinder 8.0, respectively. Each metabolite in every sample was carefully checked during peak area extraction to make sure that right peaks were extracted.

### 4.5. Data Analysis

The missing values in the sample were filled with the minimum value of each metabolite detected in other samples with the same treatment, assuming that the null value was due to the concentration of the metabolite in some samples being less than the detection limit of the instrument [53]. The peak area of each sample was divided by the median value of the compound across all samples, and the relative value was obtained for later data analysis. Log2 was used to normalize the data to a standard normal distribution, and then, a *t*-test was used to calculate in Excel 2010. A *p*-value of <0.05 was considered statistically significant. Principle component analysis was performed with SIMCA-P version 13.0, and the scaling type was “UV”. Metabolic pathways diagrams were obtained from the Kyoto Encyclopedia of Genes and Genomes (https://www.kegg.jp/ (accessed on 9 July 2022)). The data of the gene expressions were obtained from WheatGene (http://wheatgene.agrinome.org/ (accessed on 9 July 2022)) [27] and were visualized with TBtools (version v.1.098696).

### 4.6. Fungistasis Test

Petri dishes with 5 mM glutamine, 5 mM proline, 5 mM γ-aminobutyric acid, and 1 mM leucine were prepared with 10 mL of PDA culture medium. Once the culture medium was solidified, 2.5 µL of *F. graminearum* strain Fg1312 solution (1 × 10^6^ conidia per mL) was dropped into the center of the culture medium as the experimental group. No solution was added to the control group. After 3 d, the results were observed, photos were taken, and the mycelial areas were determined [57].

### 4.7. qRT-PCR

TRIzol reagent (Invitrogen, 15596018, CA, USA) was used to extract total RNA from the plant samples according to the manufacturer’s instructions. The reverse transcriptase kit (Takara, RR047A, Dalian, China) synthesized cDNA from 2 μg total RNA. qRT-PCR was performed using the Roche Thermal Cycler 96 with the TB Green^®^ Premix Ex Taq™ II reagent (Takara, RR820B, Dalian, China). Wheat *tubulin* gene was used as the internal control for analyzing metabolism-related gene expression. The PCR protocol consisted of DNA denaturation at 95 °C for 5 min, then 42–45 cycles of denaturation at 95 °C for 10 s, annealing at 56 °C for 15 s, and elongation at 72 °C for 15 s. Three biological replicates were performed. Primers used in this study were listed in Supplementary Table S5.

## 5. Conclusions

FHB is one of the most widespread and devastating diseases affecting wheat crops worldwide. Different wheat varieties possess different levels of resistance to *F. graminearum.* In order to explore this reason, we identified 365 metabolites with variable quantities in the three tested varieties. Flavonoids, hydroxycinnamate derivatives, amino acids and derivatives, carbohydrates, lipids, and a new group of nucleotides showed major changes in their expression in response to fungal infection in AN8455, YM158, and SM3. In the fungistasis test, Phenylalanine and malate suppressed the growth of *F. graminearum* mycelium, which indicate their possible future use as the green fungistatic agent in the resistance to FHB. Furthermore, some key enzymes regulating these metabolites were upregulated after infection. These results preliminarily explain the reasons for the differences in the resistances of different wheat varieties and provide new insights into the resistance mechanism of wheat.

## Figures and Tables

**Table 1:** List of significantly changed metabolites in all three varieties post *Fg* inoculation.

Compound Name	Class	Ratio (*Fg* vs. Water)		
		AN8455	YM158	SM3
agmatine	Amines	3.22	5.21	4.53
O-acetyl-L-serine	Amino acids and derivatives	3.26	1.67	1.41
S-ribosylhomocysteine	Amino acids and derivatives	2.07	3.55	3.06
cystine	Amino acids and derivatives	1.74	3.35	3.18
phenylalanine	Amino acids and derivatives	3.34	2.60	3.28
S-glutathionyl-cysteine	Amino acids and derivatives	4.08	3.33	3.28
N-acetylisoleucine	Amino acids and derivatives	7.58	5.54	3.86
spermidine	Amino acids and derivatives	3.54	8.71	4.13
N-acetylleucine	Amino acids and derivatives	11.15	5.21	5.04
homomethionine	Amino acids and derivatives	5.49	9.42	5.19
N-acetylmethionine	Amino acids and derivatives	7.06	9.67	7.27
2,6-diaminopimelic acid	Amino acids and derivatives	16.24	10.78	9.24
tryptophan	Amino acids and derivatives	2.68	3.96	9.42
5-methoxytryptamine	Amino acids and derivatives	23.94	16.45	95.10
hydroxybenzoic acid hexose II	Benzene derivatives	3.05	2.55	1.65
hydroxybenzoic acid hexose I	Benzene derivatives	1.59	1.45	1.69
kynurenate	Benzene derivatives	6.85	13.87	3.73
methyl-alpha-glucopyranoside	Carbohydrates	0.41	0.34	0.55
maleic acid	Carbohydrates	1.73	1.87	1.38
fumarate	Carbohydrates	1.73	1.96	1.44
malate	Carbohydrates	1.80	2.08	1.46
beta-hydroxypyruvate	Carbohydrates	1.96	2.35	1.61
fructose	Carbohydrates	1.63	1.83	1.62
succinate	Carbohydrates	3.62	2.14	2.21
propionic acid	Carbohydrates	3.09	1.87	2.30
threonate	Carbohydrates	3.69	3.07	2.38
pantothenic acid hexose	Cofactors	2.73	2.52	2.55
nicotinamide	Cofactors	3.58	6.49	4.85
isonicotinate	Cofactors	2.61	5.12	5.36
nicotinate	Cofactors	12.20	14.19	5.45
astilbin III	Flavonoids	2.19	3.42	1.93
naringenin	Flavonoids	12.55	2.89	3.53
feruloylquinic acid II	Hydroxycinnamate derivatives	1.63	1.54	1.33
caffeic acid hexose I	Hydroxycinnamate derivatives	2.34	1.53	1.61
cinnamic acid	Hydroxycinnamate derivatives	2.88	2.17	2.84
feruloylquinic acid III	Hydroxycinnamate derivatives	5.70	3.27	2.93
quinate	Hydroxycinnamate derivatives	4.28	2.97	3.29
o-coumaric acid	Hydroxycinnamate derivatives	7.74	5.41	3.71
coumaric acid hexoside I	Hydroxycinnamate derivatives	17.02	10.38	4.72
feruloylquinic acid-hexoside I	Hydroxycinnamate derivatives	13.24	5.94	6.92
Indole-3-acetaldehyde	Indole derivatives	36.12	38.41	26.02
3-hydroxy-3-methyl-glutaric acid	Lipids	2.22	1.52	1.42
azelaic acid	Lipids	5.25	2.69	1.60
1-LysoPE(16:0)	Lipids	4.03	3.92	2.13
1-LysoPC(16:0)	Lipids	3.77	3.09	2.14
1-LysoPE(18:1)	Lipids	3.88	5.42	2.44
3-beta-D-Galactosyl-sn-glycerol	Lipids	8.33	3.67	2.77
2-LysoPE(16:0)	Lipids	5.41	6.36	3.06
1-LysoPE(18:3)	Lipids	6.96	6.93	3.22
2-LysoPC(16:0)	Lipids	5.62	6.52	3.88
2-palmitoylglycerol	Lipids	4.96	4.47	3.95
1-LysoPC(18:3)	Lipids	11.28	8.41	4.15
hydroxyoctadecanedioic acid II	Lipids	20.66	3.32	5.83
LPG(16:0)	Lipids	16.97	13.30	7.10
homovanillic acid hexose II	Monoaromatics	1.45	2.08	1.42
guaiacol hexose-pentose II	Monoaromatics	3.07	2.63	1.65
guaiacol hexose-pentose I	Monoaromatics	1.78	4.54	3.68
homovanillic acid hexose III	Monoaromatics	49.11	48.28	34.02
5′-deoxy-5′-(methylthio)adenosine	Nucleotides	1.73	2.50	1.80
adenosine	Nucleotides	6.63	11.06	3.42
succinyladenosine	Nucleotides	8.74	7.38	7.10
inosine	Nucleotides	19.65	7.17	7.80
thymidine	Nucleotides	28.04	33.95	19.80
adipic acid	Organic acids and derivatives	9.97	5.24	2.50
dehydrophaseic acid hexose	Others	1.44	1.66	1.49
3-indoleacetic acid	Plant hormone	17.24	184.78	96.02
jasmonic acid	Plant hormones	49.43	35.26	35.14
abscisic acid hexose ester	Plant hormones derivatives	75.40	23.96	14.82

Ratios of relative metabolite levels between *Fg* and mock of the same variety. The *p*-values are available in Supplementary Table S1.

**Table 2:** Antifungal and immunity-associated metabolites.

Compound Name		Class	Ratio (*Fg* vs. Water)		
			AN8455	YM158	SM3
Antifungal metabolites	Arginine	Amino acids and derivatives	0.61	**0.52**	**0.58**
	Phenylalanine	Amino acids and derivatives	**3.34**	**2.6**	**3.28**
	Spermidine	Amino acids and derivatives	**3.54**	**8.71**	**4.13**
	Tryptophan	Amino acids and derivatives	**2.68**	**3.96**	**9.42**
	Benzoic acid	Benzene derivatives	1.25	1.05	**1.22**
	Glucosamine	Carbohydrates	**0.81**	1.06	**1.22**
	Maleic acid	Carbohydrates	**1.73**	**1.87**	**1.38**
	Fumarate	Carbohydrates	**1.73**	**1.96**	**1.44**
	Fructose	Carbohydrates	**1.63**	**1.83**	**1.62**
	Nicotinamide	Cofactors	**3.58**	**6.49**	**4.85**
	Riboflavin	Cofactors	**8.64**	**4.94**	2.36
	Tricin	Flavonoids	1.36	**1.69**	**0.48**
	Kaempferol-3-O-glucoside II	Flavonoids	1	**1.08**	**1.29**
	Cinnamic acid	Hydroxycinnamate derivatives	**2.88**	**2.17**	**2.84**
	Phosphocholine	Lipids	1.08	**1.7**	**1.2**
	Uridine	Nucleotides	**3.85**	**4.24**	**3.25**
Immunomodulatory metabolites	Agmatine	Amines	**3.22**	**5.21**	**4.53**
	Valine	Amino acids and derivatives	1.13	**1.2**	**1.15**
	Proline	Amino acids and derivatives	1.18	1.63	**1.5**
	Argininosuccinate	Amino acids and derivatives	1.47	**3.09**	**1.78**
	Gamma-aminobutyrate	Amino acids and derivatives	5.01	3.7	**3.03**
	Glutamic acid	Amino acids and derivatives	**0.46**	0.81	**1.15**
	Glutamine	Amino acids and derivatives	**0.46**	0.67	0.75
	Amino oxononanoic acid	Amino acids and derivatives	0.83	**1.41**	0.79
	Aspartate	Amino acids and derivatives	**0.5**	0.88	1.21
	Leucine	Amino acids and derivatives	**5.04**	**2.51**	1.22
	Asparagine	Amino acids and derivatives	**0.35**	0.75	1.08
	Pipecolic acid I	Amino acids and derivatives	0.58	**2.76**	0.92
	M-salicylic acid	Benzene derivatives	1.08	1.15	**1.12**
	Succinate	Carbohydrates	**3.62**	**2.14**	**2.21**
	Raffinose	Carbohydrates	**0.57**	**0.43**	0.75
	Pyruvate	Carbohydrates	**1.92**	1.09	1.18
	Glucose 6-phosphate	Carbohydrates	**0.64**	1.53	1.35
	Guanosine	Nucleotides	2.74	**3.88**	**2.93**
	Guanine	Nucleotides	**6.62**	**10.89**	3.03
	Xanthine	Nucleotides	**3.8**	1.38	1.1
	Jasmonic acid	Plant hormones	**49.43**	**35.26**	**35.14**

Ratios of relative metabolite levels between *Fg* and mock of the same variety. The bold values represent significantly different metabolic levels between *Fg* and mock samples (*p*-values < 0.05). The *p*-values are available in Supplementary Table S1.

## Data Availability

All data used or analyzed in this study are included in this published article and its Supplementary Materials.

## Supplementary Materials

The following supporting information can be downloaded at: https://www.mdpi.com/article/10.3390/ijms24043214/s1.
