# Supplementary figures and images for: Investigating the Resistance Mechanism of Wheat Varieties to Fusarium Head Blight Using Comparative Metabolomics
Source: Int J Mol Sci. 2023 Feb 6;24(4):3214. doi: 10.3390/ijms24043214 (PMC9960685; doi:10.3390/ijms24043214)

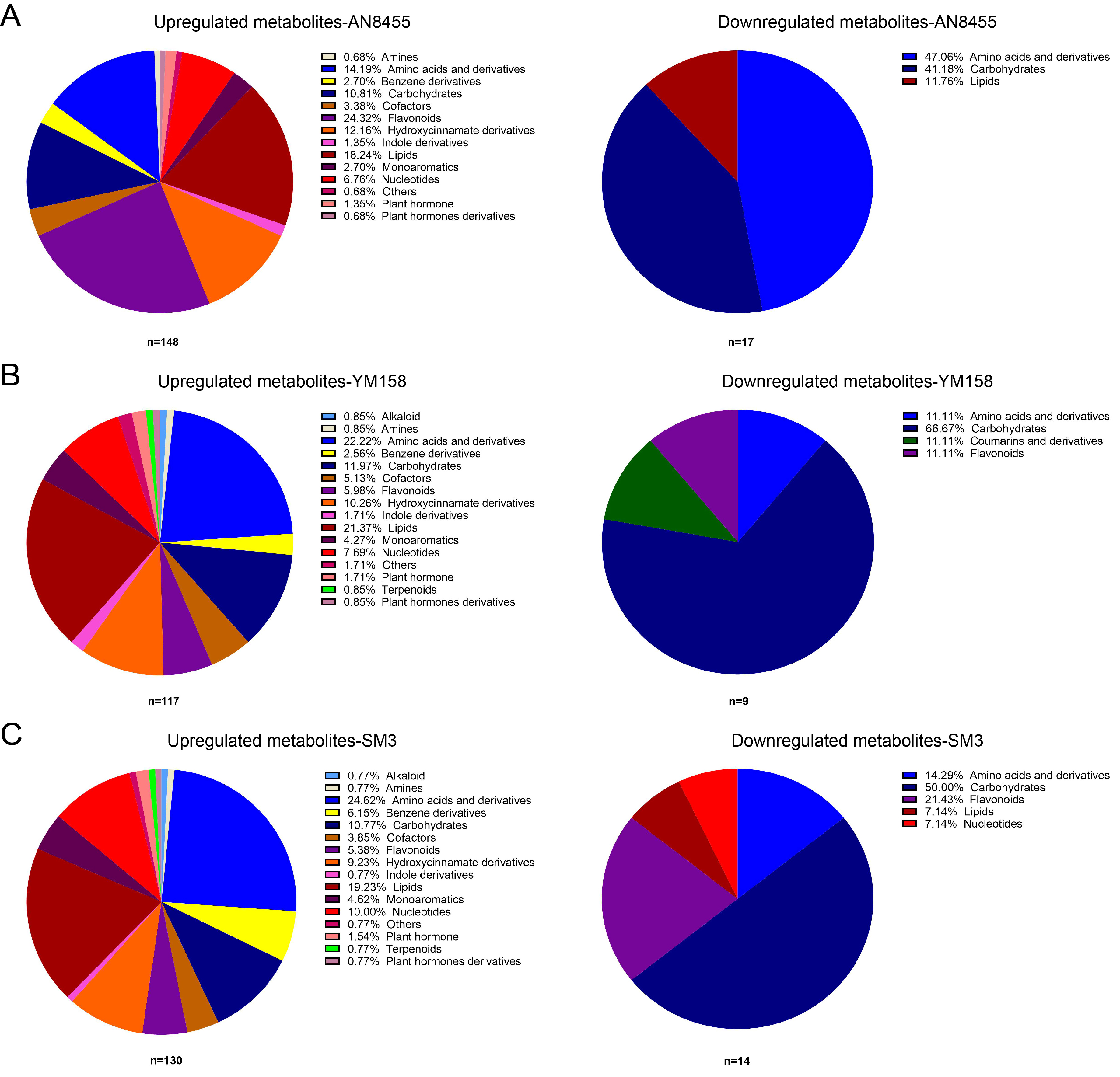

Supplement: Supplementary file 1 [file ijms-24-03214-s001.zip › ijms-2090828-supplementary/SFIG-1.jpg]

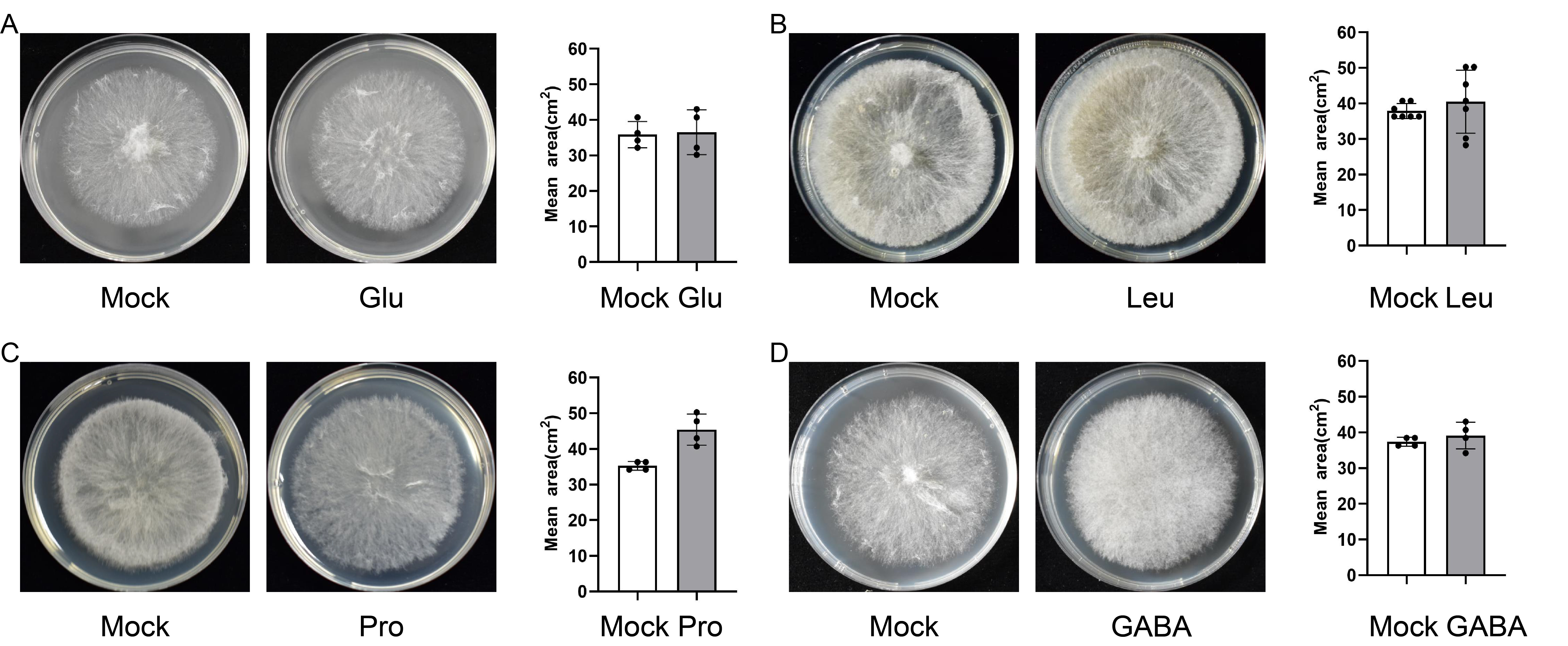

Supplement: Supplementary file 1 [file ijms-24-03214-s001.zip › ijms-2090828-supplementary/SFIG-2.jpg]
